# Evaluation of SARS-CoV-2-Specific T-Cell Activation with a Rapid On-Chip IGRA

**DOI:** 10.1021/acsnano.2c09018

**Published:** 2023-01-03

**Authors:** Bo Ning, Sutapa Chandra, Juniper Rosen, Evan Multala, Melvin Argrave, Lane Pierson, Ivy Trinh, Brittany Simone, Matthew David Escarra, Stacy Drury, Kevin J. Zwezdaryk, Elizabeth Norton, Christopher J. Lyon, Tony Hu

**Affiliations:** † Center for Cellular and Molecular Diagnostics, Tulane University School of Medicine, New Orleans, Louisiana 70112, United States; ‡ Department of Biochemistry and Molecular Biology, Tulane University School of Medicine, New Orleans, Louisiana 70112, United States; § Department of Microbiology & Immunology, Tulane University School of Medicine, New Orleans, Louisiana 70112, United States; ∥ Department of Physics and Engineering Physics, Tulane University, New Orleans, Louisiana 70118, United States; ⊥ Department of Psychiatry, Tulane University, New Orleans, Louisiana 70112, United States; # Tulane Brain Institute, Tulane University, New Orleans, Louisiana 70112, United States

**Keywords:** T-cell response, COVID-19, IGRA, COVID-19 vaccine, rapid test, whole blood assay

## Abstract

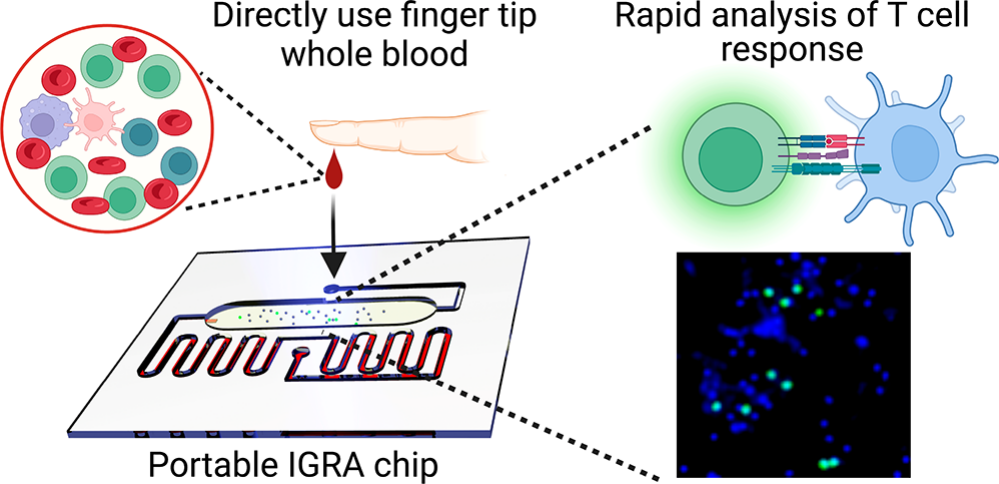

Interferon-gamma release assays (IGRAs) that measure pathogen-specific T-cell response rates can provide a more reliable estimate of protection than specific antibody levels but have limited potential for widespread use due to their workflow, personnel, and instrumentation demands. The major vaccines for SARS-CoV-2 have demonstrated substantial efficacy against all of its current variants, but approaches are needed to determine how these vaccines will perform against future variants, as they arise, to inform vaccine and public health policies. Here we describe a rapid, sensitive, nanolayer polylysine-integrated microfluidic chip IGRA read by a fluorescent microscope that has a 5 h sample-to-answer time and uses ∼25 μL of a fingerstick whole blood sample. Results from this assay correlated with those of a comparable clinical IGRA when used to evaluate the T-cell response to SARS-CoV-2 peptides in a population of vaccinated and/or infected individuals. Notably, this streamlined and inexpensive assay is suitable for high-throughput analyses in resource-limited settings for other infectious diseases.

## Introduction

Interferon-gamma release assays (IGRAs) measure T-cell activation in response to pathogen-specific peptides as a surrogate for an individual’s potential immune response to a pathogen of interest and an estimate of protective immunity. IGRAs measure either the absolute amount of interferon gamma (IFNγ) released after antigen stimulation by ELISA or the number of cells that secrete IFNγ after this stimulation. The second approach uses an enzyme-linked immunosorbent spot (ELISpot) assay to detect IFNγ released and bound in proximity to activated T-cells immobilized on a detection membrane. Each IGRA approach has drawbacks that limit their feasibility for routine use in large populations to estimate the efficacy of vaccination or previous infection in conferring protective immune responses. Both ELISA and ELISpot IGRAs require whole blood, which must be processed within 8–14 h after collection to obtain reliable IGRA results. ELISpot assays provide a direct indication of the relative number of antigen-responsive T-cells but are more technically demanding than ELISA-based IGRAs since they analyze the IFNγ response in cultured and stimulated peripheral blood mononuclear cells (PBMCs) isolated and quantify colorimetric spots produced by activated T-cells. However, both ELISpot and ELISA-based IGRAs can be technically demanding and are typically performed at central laboratories, so sample shipping logistics can be a limiting factor in assay performance.

Given the continuing emergence of new SARS-CoV-2 variants of concern (VOCs), approaches are needed to estimate the immune protection individuals may have against a specific VOC at various time points after vaccination or infection by a different virus strain. This information is of critical importance for evaluations of vaccine effectiveness that inform vaccination guidelines and public health decisions. It can also be used to identify vulnerable populations or individuals who require further precautions or interventions, including additional vaccine doses. Studies have shown that immune responses to SARS-CoV-2 produced by vaccinated and previously infected individuals offer reduced protection against SARS-CoV-2 VOCs,^1−3^ including B.1.1.7 (Alpha),^4^ B1.351 (Beta),^5^ P.1 (Gamma),^6^ B.1.617.2 (Delta),^4^ and B.1.1.529 (Omicron).^7^

Better understanding of how immune responses to SARS-CoV-2 VOCs change with time is essential to estimate their role in the durability of vaccine-mediated protection against these variants. However, the kinetics of antibody responses postinfection or postvaccination vary among different populations,^8−10^ and this data has shown limited clinical value when used to monitor vaccine efficacy over time.^11^ SARS-CoV-1 and MERS studies have also reported that T-cell responses persist much longer than antibody responses, including in the absence of detectable antibody responses.^12−14^ Evidence also indicates that SARS-CoV-2-specific T-cell responses remain active after neutralizing antibody titers decrease^14−16^ and can be observed in the absence of detectable specific antibodies.^17^ For example, immunocompromised individuals can exhibit inadequate seroconversion rates and neutralizing antibody responses following SARS-CoV-2 vaccination^18−22^ but still demonstrate a significant virus-specific T-cell response,^22^ including a strong response to Omicron. Notably, Omicron can evade specific neutralizing antibodies^2,3,23^ but can still activate T-cell responses induced by prior vaccination or infection,^2,24−26^ with one study indicating that 70–80% of the vaccine-induced CD4 and CD8 T-cell response to the reference strain spike protein was retained for Omicron.^27^ Several studies have employed IGRAs to evaluate T-cell responses in vaccinated individuals and SARS-CoV-2 patients,^28−38^ and the analysis of T-cell responses to emerging SARS-CoV-2 VOCs may allow rapid evaluation of vaccine efficacy to and inform the need for additional vaccine doses or variant-specific vaccines.

Streamlined IGRAs with reduced technical demands and decreased performance times are required for rapid IGRA analyses. Traditional assays that analyze antigen-specific T-cell responses typically have lengthy workflows and may require substantial liquid handling, technical expertise, or specialized equipment. Microfluidic devices can simplify assay workflows, reduce required sample volumes and reagent costs, and minimize operator effort and expertise requirements. They can also reduce assay variation by automating key steps that may be particularly susceptible to minor differences in sample handling, such as cell capture. Microfluidic approaches have thus been employed for several COVID-19 diagnostic assays.^39−42^ We thus hypothesized that a microfluidic IGRA could be developed to permit high-throughput analysis of the T-cell response to SARS-CoV-2 antigens.

Here we describe the development and characterization of a microfluidic ELISpot IGRA test integrated with a nanolayer of polylysine suitable for rapid analysis of the T-cell response to SARS-CoV-2 peptides. Our chip combines sample capture, cell fixation and permeabilization, antibody labeling, and all wash steps in the integrated microfluidic device to significantly reduce user effort and sources of variability, while assay readout reduces the required sample volume to reduce reagent requirements and the assay completion time. We found that results from this assay were comparable to those from flow cytometry and conventional ELISpot analyses, when used to evaluate responses of individuals who had or had not been vaccinated against or infected with SARS-CoV-2. This 5 h assay analyzes fingerstick blood samples (∼25 μL) using a workflow for the sample handling and equipment requirements in a format readable by a cellphone microscope to permit its use in resource-limited areas.

## Results/Discussion

### Design and Development of a Slide-Based ELISpot IGRA System

**Scheme 1:**
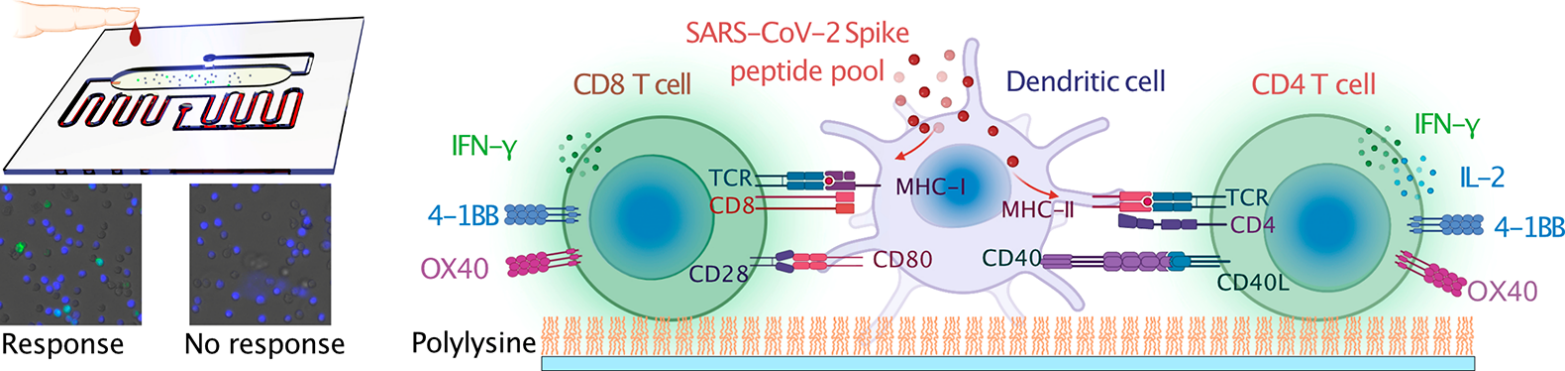
Microfluidic Chip IGRA Test Fingertip whole blood can be directly used for SARS-CoV-2 spike protein peptide pool stimulation and positive T-cell detection (left). All PMBC will be captured by the polylysine nanolayer coated on the glass surface, and then, antigen-presenting cells such as dendritic cells will present peptides to CD4 or CD8 T-cells. IFNγ in activated T-cells will be stained with fluorescent antibody and imaged with this microfluidic chip.

ELISpot assays have greater procedural and equipment requirements than ELISA-based IGRAs but are more readily adapted to a microfluidic assay workflow, since they require fewer liquid handling steps in certain assay designs. The ELISpot microfluid workflow can be broken down into a few basic steps (blood collection, T-cell stimulation with pathogen-specific peptides, and the capture, staining, and analysis of activated T-cells), most of which can be accomplished on a microfluidic chip to simplify the ELISpot workflow (Scheme 1, Figure S1).

**Figure 1:**
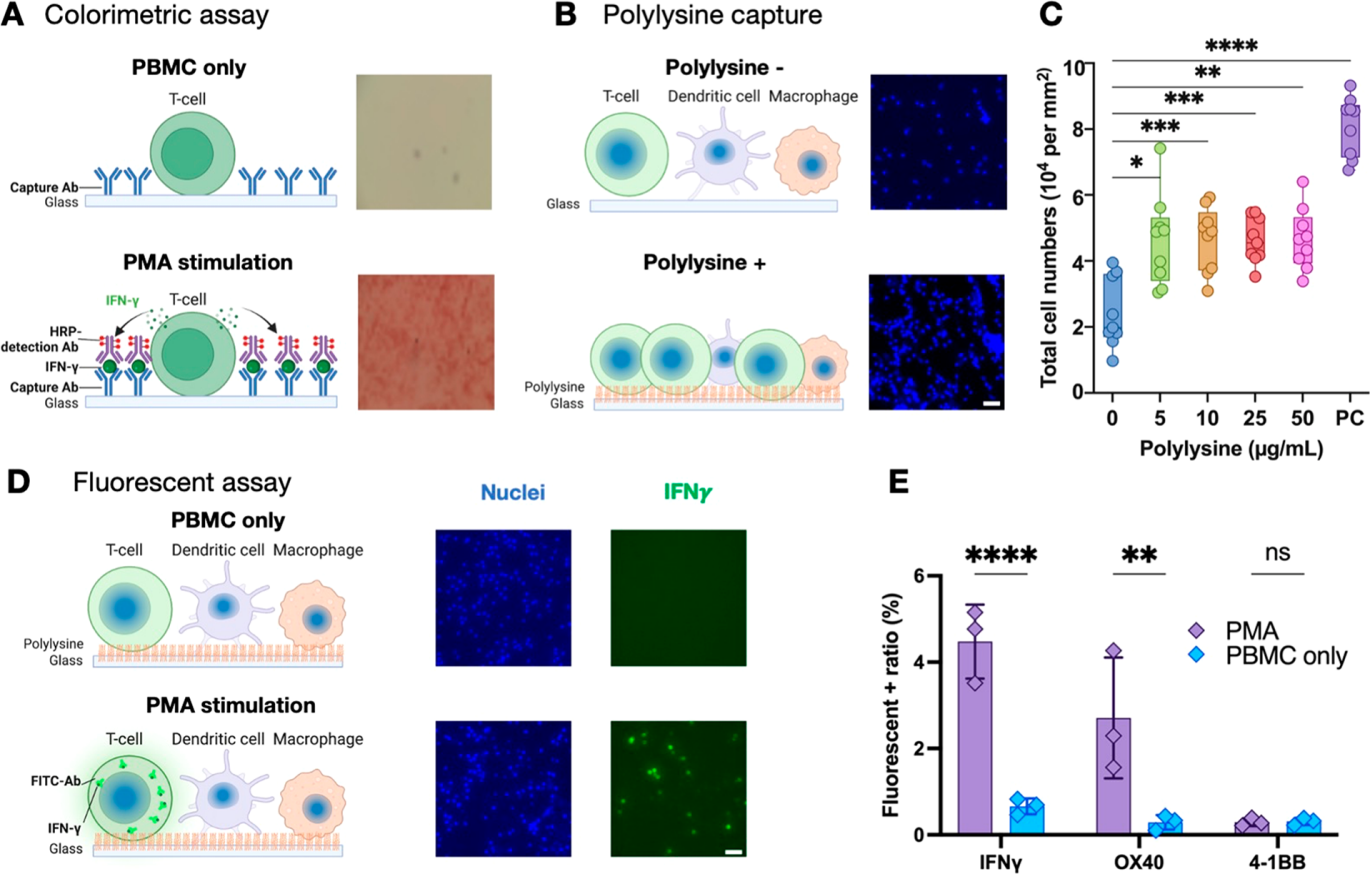
Slide-based PBMC activation analyses. (A) Thawed PBMC aliquots stimulated with or without PMA/ionomycin were cultured for 24 h in glass bottom wells coated with IFNγ-specific antibody then incubated with a biotinylated secondary antibody, streptavidin-HRP, and a chromogenic (red) HRP substrate. (B–E) PBMCs (∼2 × 10^5^) were seeded on microplate wells coated with and without polylysine and stained with Hoechst 33342 to quantify the cell density of captured cells, or (D, E) induced with PMA/ionomycin for 4 h, stained with Hoechst 33342 (B, C) and specific antibodies to IFNγ, OX40, and 4-1BB (D, E), after which total cell numbers and activated T-cell percentages were quantified using a fluorescent plate reader. Positive control (PC) wells were not washed to remove nonadherent or weakly adherent cells. One-way two-sided parametric ANOVAs with Tukey’s post-test were performed to analyze differences between the polylysine-coated and uncoated well values and (E) PMA-stimulated and unstimulated well values. White size bars indicate 75 μm. Data indicate mean ± SD; *, *p* < 0.05; **, *p* < 0.01; ***, *p* < 0.001; ****, *p* < 0.0001; ns, no significant difference when analyzed by two-sided Mann–Whitney U-test. The schematics in A, B and D were created with BioRender.com.

Microfluidic chips are routinely constructed on glass or plastic substrates, while ELISpot assays usually employ nitrocellulose or polyvinylidene fluoride membranes that have high binding capacity for IFNγ capture antibodies used in its ELISA and provide good contrast for the detection of the chromogenic signal produced upon recognition by IFNγ captured around activated T-cells.^43−46^ We therefore evaluated whether we could detect the ELISpot chromogenic substrate on assay slides conjugated with IFNγ capture antibodies after their incubation with stimulated PBMC samples. We observed a chromogenic signal on slides incubated with PMA-stimulated PBMC samples but not slides incubated with unstimulated PBMC samples, but the weak and diffuse nature of this signal did not permit accurate quantification of activated cell numbers (Figure 1A). This may have been due to weak binding of the chromogenic substrates to the surface of these slides that could have reduced localized binding and promoted the loss of surface bound chromogen during the post-ELISpot wash step. The scheme was created with BioRender.com.

We therefore next attempted to modify our assay readout approach to directly detect the activated T-cells among the PBMCs bound to the assay slide following antigen stimulation. PBMC binding was improved by precoating assay slides with polylysine (Figure 1B), which is a widely used hydrogel^47^ to enhance cell capture, including lymphocyte capture, by forming electrostatic interactions with anionic molecules on their plasma membranes.^48,49^ According to different solid surfaces and buffer conditions, polylysine will form nanolayers on a silica glass surface.^50^ A titration study found 5 μg/mL polylysine, which is a ∼10 nM nanolayer,^51^ was sufficient to maximize PBMC capture, doubling the capacity of untreated slides (4.5 × 10^4^ versus 2.2 × 10^4^ PBMC/mm^2^) to capture ∼58% of the input PBMCs (∼7.7 × 10^4^ PBMC/mm^2^) (Figure 1C). Next, activated T-cells were detected by hybridizing fixed and permeabilized PBMC samples with an IFNγ-specific fluorescent antibody, which detected strong activation signals only in the PBMC samples preactivated with phorbol myristate acetate (PMA) and ionomycin (Figure 1D). IFNγ is routinely used as a marker of T-cell activation, but its detection in our modified IGRA requires PBMC fixation and permeabilization to allow the assay antibody to recognize its intracellular expression, which may reduce cell numbers and increase assay background. We therefore also evaluated two surface markers of T-cell activation that can be detected without cell permeabilization, OX40^52,53^ and 4-1BB,^54,55^ to evaluate their potential utility as activation markers. The OX40 and 4-1BB signal was moderately lower than the IFNγ signal in unstimulated PBMC (Figure S2); however, the 4-1BB signal did not change after a 24 h PMA/ionomycin stimulation, and the increase in OX40 signal was lower and more variable than the corresponding IFNγ signal increase (Figure 1E), leading us to select IFNγ as the T-cell activation marker for all future experiments.

**Figure 2:**
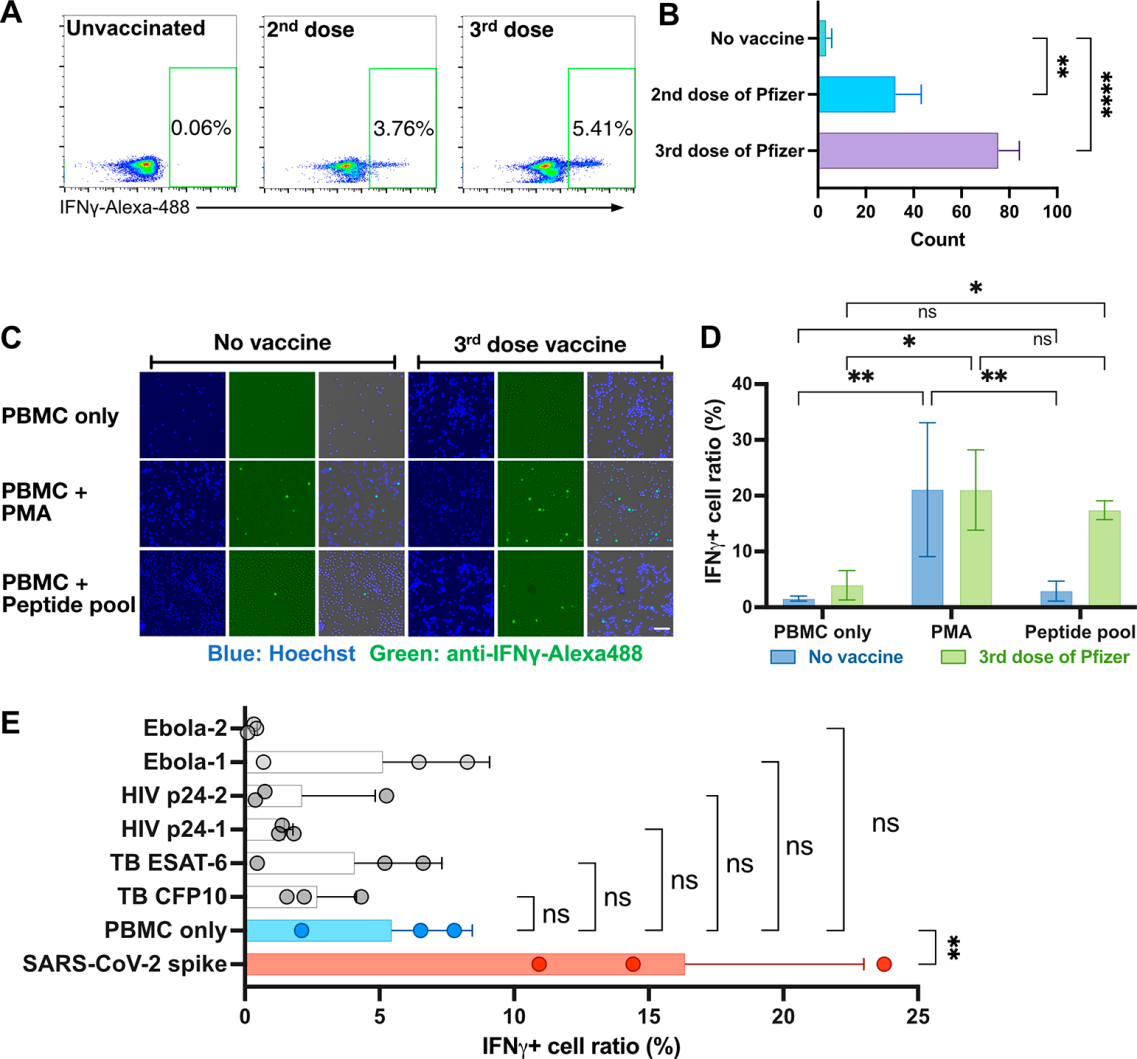
T-cell activation with SARS-CoV-2 spike peptide pool. (A, B) Cryopreserved PBMCs from individuals who had received zero (unvaccinated), two, or three SARS-CoV-2 vaccine doses were simulated by incubation with a SARS-CoV-2 peptide pool for 24 h, after which IFNγ+ cells were detected by (A) flow cytometry or (B) ELISpot. (C) Representative images and (D) quantification results from freshly isolated PBMCs from individuals who were unvaccinated and or had received three vaccine doses after 24 h incubation with or without a SARS-CoV-2 peptide pool. (E) Summary of the IFNγ+ cell ratios detected in PBMCs of SAR-CoC-2-vaccine recipients (three doses) following 24 h incubation with peptides from SARS-CoV-2, the *M. tuberculosis* (*Mtb*) CFP-10 and ESAT-6 proteins, HIV-1 p24, or Ebola VP40 protein. Data indicate mean ± SD; symbols denote *p* < 0.05 (*), *p* < 0.01 (**), *p* < 0.001 (***), or nonsignificant (ns) differences between indicated groups by (C) one-way parametric ANOVA with Tukey’s post-test or (D, E) Mann–Whitney U-test.

We next evaluated the ability of this approach to detect an antigen-specific T-cell activation response. To do so, we first evaluated the ability of an SAR-CoV-2 peptide pool to induce a T-cell activation response in PBMCs isolated from individuals who were unvaccinated with no history of infection or who had received three doses of a SARS-CoV-2 RNA vaccine to determine a baseline for this activation response, using two assay approaches that measure the T-cell activation percentage similar to our proposed assay. Flow cytometry analysis of SARS-CoV-2-responsive T-cells in PBMC samples of vaccinated and unvaccinated/uninfected individuals detected a low response rate (0.01%) 24 h after peptide stimulation in the unvaccinated/uninfected group (Figure 2A) and revealed that this rate progressively increased in individuals who had received two and three vaccine doses (3.76% and 5.41%, respectively). Similar results were observed when these samples were analyzed with a standard ELISpot IGRA, which, respectively, detected an average of 5, 36, and 77 antigen-responsive T-cells in samples from these three groups (Figure 2B).

We next evaluated the IFNγ+ cell ratios detected by our microfluidic ELISpot assay 24 h after PBMC samples, isolated from individuals who were unvaccinated/uninfected or had received three vaccine doses, were incubated with or without a pool of SARS-CoV-2-derived peptides or PMA/ionomycin. No significant T-cell activation was detected in unstimulated PBMCs of unvaccinated/uninfected individuals; PMA/ionomycin stimulation produced robust activation responses in both groups, and the SARS-CoV-2 peptide pool induced T-cell activation only in the vaccinated group (Figure 2C,D). Further, this activation response was found to be pathogen-specific, since the IFNγ+ cell ratios detected when PBMCs of SARS-CoV-2 vaccine recipients were incubated with peptides derived from other pathogens to which they had no previous exposure (e.g., TB and HIV) were not different from those measured with unstimulated PBMCs (Figure 2D).

### Microfluidic Chip ELISpot IGRA Performance

**Figure 3:**
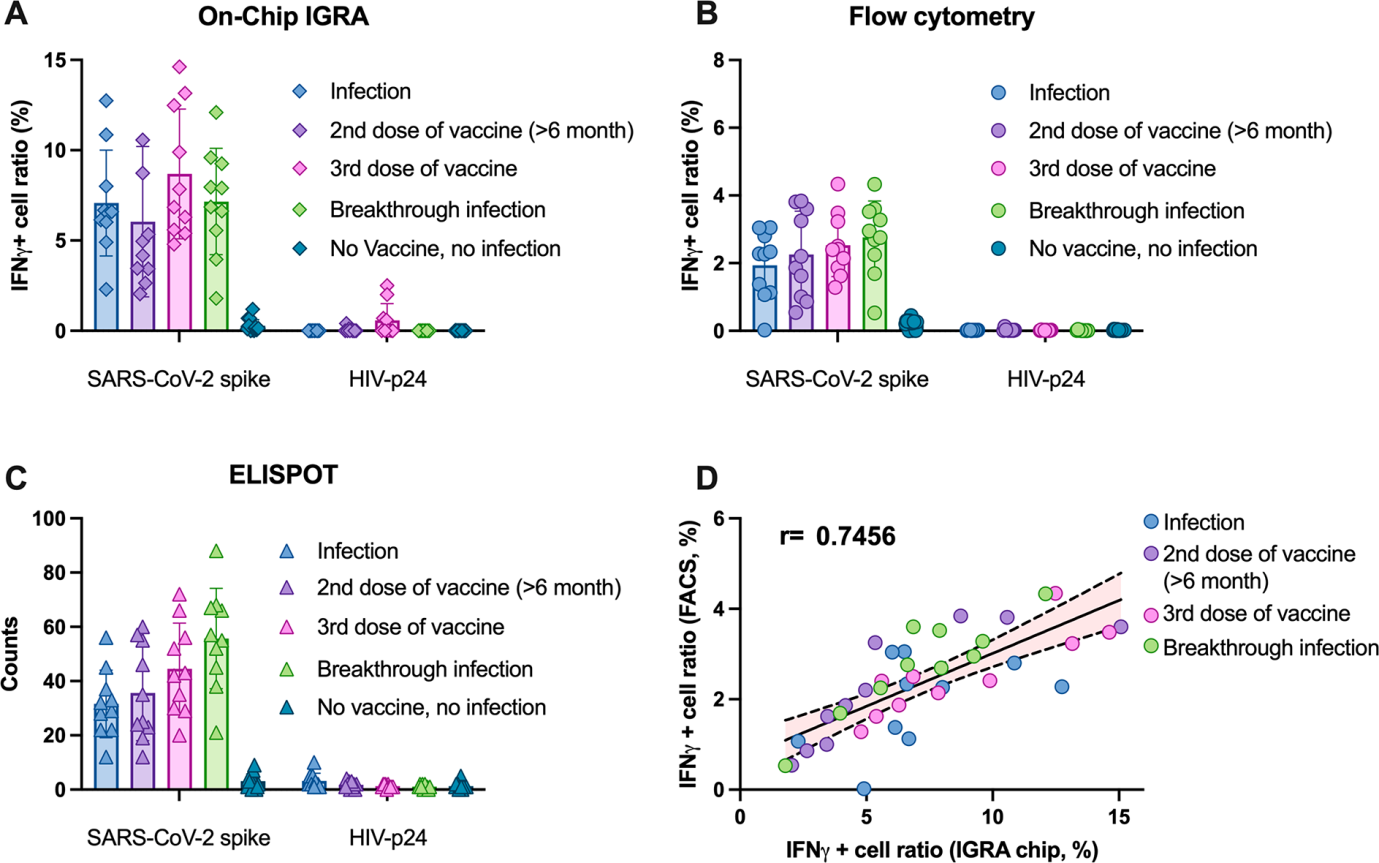
Evaluation of on-chip ELISpot assay results in vaccinated HIV– individuals. (A) On-chip ELISpot, (B) flow cytometry, and (C) ELISpot assay produced after stimulating PBMCs isolated from individuals without a history of HIV infection who had received three vaccine doses with SARS-CoV-2 spike or HIV-1 p24 (nonspecific control) peptides. (D) Correlation of flow cytometry and on-chip ELISpot data. Data indicate mean ± SD; symbols indicate *p* < 0.05 (*), *p* < 0.01 (**), or ns (nonsignificant) differenced by two-sided Mann–Whitney U-test.

These cell capture, stimulation, and analysis steps were then combined into an integrated on-chip microfluidic assay procedure to evaluate overall assay performance. Microfluidic chip wells coated with polylysine were loaded with ∼2 × 10^6^ PBMCs, cultured for 24 h with or without the target peptide, and then fixed, permeabilized, and incubated with Hoescht 33342 and an IFNγ-specific fluorescent antibody, after which lab images of labeled-cells were captured by a fluorescence microscope and analyzed to evaluate PBMC activation. IFNγ+ cell percentages detected in unstimulated PBMC samples in this analysis were markedly lower than previously detected, as was the percentage of cells stimulated upon incubation with the SARS-CoV-2 peptide pool, which did not differ among the vaccinated and/or infected groups (Figure 3A). ELISpot and flow cytometry analyses of these samples produced similar results but revealed modest progressive increases among these groups according to the number of antigen exposure events (Figure 3B,C and Figure S3). ELISpot and ELISA-based IGRA do not exhibit a strong correlation, unlike ELISpot and flow cytometry assay data, which demonstrates good correlation, albeit with substantial variation (Figure 3D). Notably, the mean percentage of IFNγ-positive cells detected by on-chip assay in all antigen-exposed groups (5.3 ± 4.2%) was higher than that determined by flow cytometry (1.8 ± 1.3%), although the results revealed good correlation, with strong correlations observed with all but the infection-based exposure group, with the slope of the correlation line of each group increasing with the number of exposure events associated with each group (Figure S4A–D). However, only a weak correlation was observed upon comparison of the on-chip and standard ELISpot data, likely due to variability introduced by differences in their respective assay procedures.

### Microfluidic IGRA Analysis of Fingerstick Whole Blood Samples

**Figure 4:**
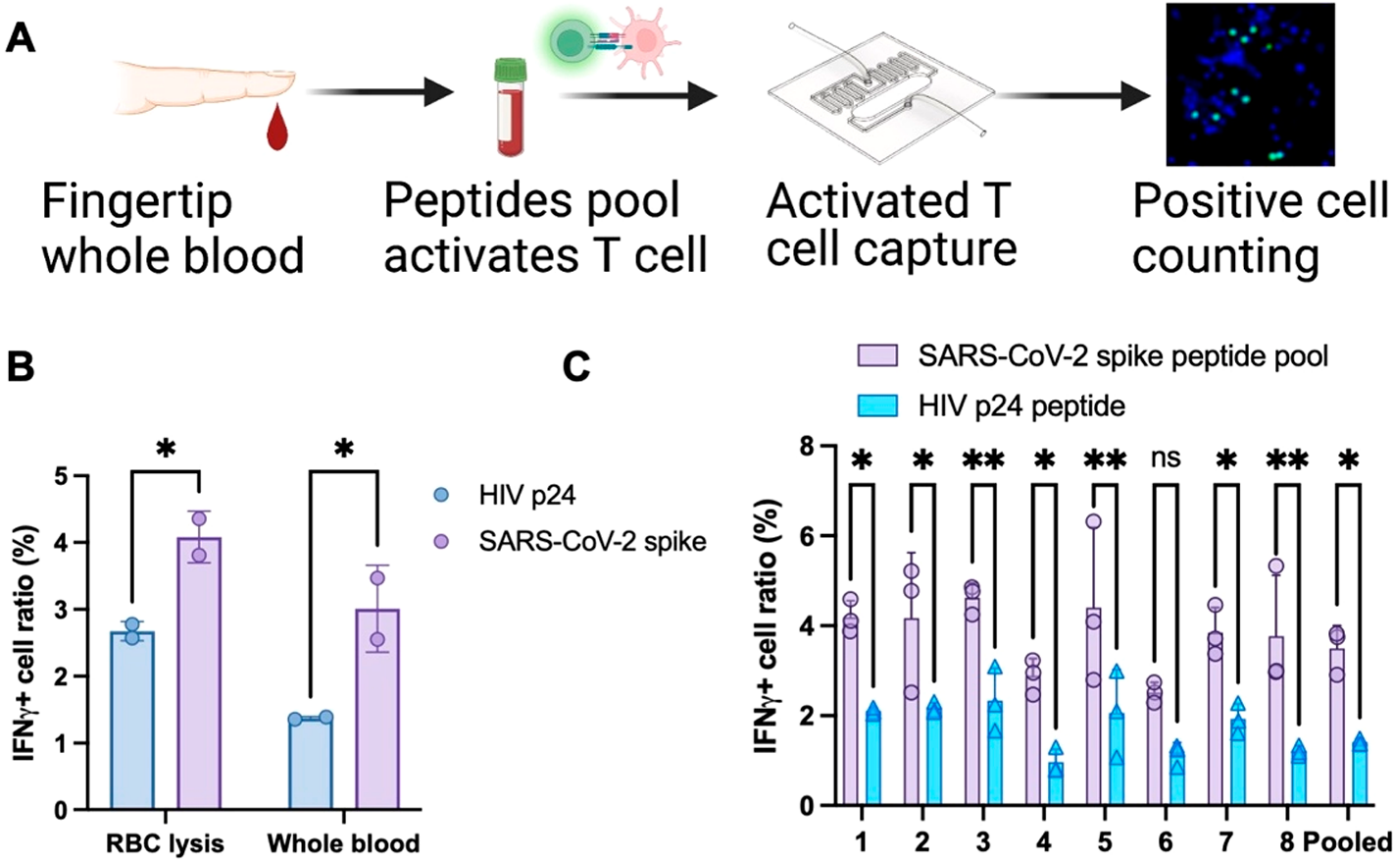
Evaluation of on-chip ELISpot assay with whole blood samples. (A) Scheme of whole blood T-cell evaluation with an on-chip ELISpot test. (B, C) On-chip ELISpot assays result from fingerstick whole blood samples (B) from one subject, with and without RBC lysis, and (C) from eight HIV negative individuals >6 months after receipt of two or three vaccine doses, without RBC lysis. Data indicate mean ± SD; symbols indicate *p* < 0.05 (*), *p* < 0.01 (**), or ns (nonsignificant) differenced by two-sided Mann–Whitney U-test. The schematic in A was created with BioRender.com.

ELISpot assays require extended culture (16 h) of PBMCs isolated from >5 mL of venous blood, which hinders their use in resource-limited settings. We therefore evaluated whether our on-chip ELISpot assay could be performed with a shorter interval (4 h) using fingerstick blood volumes (∼25 μL) with or without an intermediate red blood cell (RBC) lysis step (Figure 4A). This analysis found that RBC lysis increased the number of captured PBMCs versus whole blood samples but also increased nonspecific T-cell activation in response to a control peptide, resulting in a corresponding signal-to-noise decrease (1.3-fold versus 2.2-fold) in samples exposed to RBC lysis (Figure 4B). Subsequent analysis of fingerstick whole blood samples collected more than six months after receipt of a third vaccine dose from individuals who had no history of HIV infection detected a similar degree of specific induction in all samples (2.4 ± 0.8-fold induction), which reached significance in all but one sample (Figure 4B). The mean IFNγ-positive cell percentage detected in this analysis (3.8%) was lower than observed in on-chip ELISpot assays performed with isolated PBMCs from similarly vaccinated individuals (Figure 3C; 9.6%); however, this was balanced by reduced sample variance.

Immunoassays that detect the presence or titer of specific antibodies to pathogen-derived factors, or the percentage or activity of T-cells that respond to these factors, provide important but divergent information that is useful in evaluating the efficacy of an individual’s potential immune response to these pathogens. Assays that detect pathogen-specific antibodies are straightforward, can be readily employed in most settings, and are thus often suitable for POC test, but may not provide a reliable picture of immunity as circulating antibody responses can wane long before the loss of inducible immunity. IGRAs are potentially useful to address this question but are not suitable for high-throughput use or use in resource-limited settings and thus are not practical for evaluating individual immune response at large scale. Here we demonstrate that a modified ELISpot IGRA can be employed to address the shortcoming of traditional ELISAs, since it can be performed using fingerstick rather than venous blood volumes; analyze whole blood, eliminating the need for sample processing to isolate PBMCs; and be read within ∼5 h of sample collection using a fluorescent microscope or plate reader. One of the challenges of traditional IGRA is the complex and time-consuming sample processing steps. Our first and essential solution is to utilize microfluidic chips to simplify overall procedures. Although microfluidic chips are used for immunoassays^56,57,40,43−46^ and point-of-care devices,^58^ they are still limited to measuring homogeneous lysate but not cell-based assays. To achieve our goal of the portable assay for cell response, we utilized ∼10 nm polylysine nanolayers to immobilize immune cells in a microfluidic chamber (Figure S1). This immobilization will first benefit the close contact of antigen-presenting cells (APC) like dendritic cells and memory CD4 or CD8 T-cells (Scheme 1, Figure 1B,C), speeding up the T-cell responses of IFNγ release. Reduced movement of cells also provides more accurate cell imaging and counting results.

An ELISpot assay approach was chosen for this analysis since this assay format measures the fraction of T-cells that is responsive to a selected pathogen-derived factor and thus provides a direct measure of the cell population available to respond to this pathogen. ELISA-based IGRAs, which are more commonly used, measure the relative degree of the cytokine response and thus integrate the number of available cells and the extent of their inducible cytokine response.

Conventional ELISpot assays analyze dye foci deposited on polyvinylidene difluoride (PVDF) membranes, but this readout approach employs a sandwich ELISA in which IFNγ released by activated cells is captured on the PVDF membrane at their position and hybridized with an enzyme-conjugated IFNγ-specific antibody to permit *in situ* conversion and binding of a colorimetric substrate. This requires multiple wash steps, careful control of incubation and reaction times, and a readout device that can capture high-magnification images illuminated with a high-intensity light source, complicating the assay workflow, increasing equipment demands, and reducing utility in resource-limited settings. In this study, we replaced this approach with an equilibrium-based intracellular staining workflow used in flow cytometry to simplify its readout procedure, which employs the codetection of nuclear staining to visualize relative cell activation rates without requiring the analysis of predetermined numbers of PBMCs (Figures 2 and 3). RBC removal was not required for this analysis since this process did not markedly reduce PBMC binding and appeared to increase nonspecific cell activation, thereby decreasing the relative degree of specific induction. However, nuclear RBCs were not included in the cell count and thus did not affect calculated cell activation percentages (Figure 4B,C).

Standard ELISpot assays evaluate the number of IFNγ-positive cells within a standard PBMC sample size. This requires the rapid isolation of viable PBMCs, which must then be counted and analyzed for viability, diluted to a standard concentration of viable cells, and then cultured overnight after exposure to an antigen. All of these requirements add complexity that renders these assays impractical for use in many settings. However, our revised ELISpot assay employs fingerstick whole blood microsamples, eliminating the need for a trained phlebotomist to perform a venous blood draw and the need to isolate PBMCs and allowing the number of IFNγ-positive and total PBMCs present in a sample to be directly measured by image analysis. Given the limited opportunity for variation in the sample collection and processing procedure, it can also be assumed that cell viability should not influence the IFNγ-positive cell percentages in this approach.

This ELISpot assay procedure eliminates most obstacles that limit the widespread use of IGRAs, but several aspects could be further optimized to improve assay performance. For example, results from this assay did not distinguish sets of samples from individuals who had different numbers of exposure events, although flow cytometry and standard ELISpot results revealed trends toward differences between these groups. This may be due to the relatively small number of cells captured on the microwell, the loss of activated T-cells during the washing step, and/or sampling bias during image capture and analysis.

Improving the precision and reproducibility of such assay measurements may be important to improve the ability to sensitively track the durability of acquired T-cell responses to specific pathogen-derived antigens and the relative amount of protective immunity retained over time. Enhanced precision could be obtained using several approaches, either alone or in combination. Cell capture was enhanced by using polylysine-coated assay wells, but this cell binding approach is not T-cell-specific and may limit the reproducibly of cell capture, culture, and retention during the assay procedure. The use of CD4- and/or CD8-specific antibodies could improve the capture and retention of T-cells induced in off-chip cell induction and staining reactions to reduce assay variability but might require titration to prevent capture cell densities from obscuring the number of total and/or positive cells present in an assay, or fabricating assay chips so that antibodies are spotted in a dispersed array. Microfluid chip assay advantages cannot be readily achieved with other workflows that employ small volumes, such as a microplate assay, since these formats require a user to carefully dispense and aspirate small volumes that may be difficult to precisely control and thus lead to assay variability from multiple sources (e.g., variable volumes, carryover effects, differences in tip placement or flow rates that may produce difference in cell capture and loss, etc.). Some of these effects could potentially be addressed with automated fluid handling devices but require the use of expensive equipment. Finally, an ideal version of this ELISpot assay would be read by an inexpensive portable device to allow analysis on-site in resource-limited settings. We have previously developed an inexpensive fluorescent smartphone microscope and app that can be used to read other chip-based assays and which could be modified to read, analyze, and report results for our current ELISpot assay. This technology could also allow the recovery of activated cells for single-cell intracellular or secreted protein analyses with minimal modifications.

## Conclusions

In summary, this modified ELISpot approach permits the rapid and inexpensively analysis of T-cell activation responses using fingerstick whole blood microsamples, without significant equipment or technical expertise. This platform should allow high-throughput analysis of T-cell responses to specific pathogen-derived antigens as a measure of potential immunity after infection or vaccination. For example, the potential resistance to new variants of these pathogens can be evaluated by altering the peptide pool to contain peptides sequences specific for these variants. Our on-chip IGRA approach also has potential as a faster, less expensive, and higher-throughput alternative to ELISpot and QuantiFERON IGRAs that are often employed to detect Mtb infections as part of the workup for TB diagnosis but that have additional constraints. For example, ELISpot IGRAs require special supplies and equipment (a scanner) and well-trained personnel to perform the PBMC isolation, cell culture, and analysis procedures necessary for this assay. QuantiFERON IGRAs require fewer resources and less training but still require expensive supplies and all the materials and technical ability required to perform an ELISA. Further, a variant of this approach could also be generated to measure memory B cell responses. Large-scale evaluation of acquired immune responses using such approaches should benefit studies designed to evaluate vaccine effectiveness for existing and emerging infectious diseases and may improve understanding of some chronic infections.

## Methods/Experimental

### Patient Population

Whole venous blood and fingerstick blood samples were obtained from a population of SARS-COV-2-infected and/or vaccinated adults enrolled in our study at New Orleans Childen’s Hospital.

Subjects or households with suspected or confirmed SARS-CoV-2 infection were recruited from the Greater New Orleans community under Tulane Biomedical Institutional Review Board (federal wide assurance number FWA00002055, under study number 2020-585). Enrolled subjects completed a study questionnaire regarding infection and demographic information and provided a blood sample.

For the fingerstick blood analysis studies, healthy SARS-COV-2-vaccinated adults aged 21–41 years were enrolled in the study following a protocol approved by the Institutional Review Board of Tulane University. Written informed consent was obtained from each participant before study participation. A SARS-SOV-2 screening questionnaire and information regarding vaccination status were also obtained. Fingertip blood samples were collected from each participant using a contact-activated lancet (BD 355594) to collect 200–400 μL of blood into lithium heparin micro blood collection tubes (BD 365965), which were then immediately processed for on-chip ELISpot analysis.

### PBMC Isolation

PBMCs were isolated from frozen leukapheresis samples (Stemcell Technologies) or whole blood samples. Venous blood samples were collected in EDTA tubes and supplemented with a 15× volume of cold (4 °C) isotonic ammonium chloride solution, mixed by inversion at room temperature for 10 min using a rotary mixer set to ∼500 rpm to allow RBC lysis, and then centrifuged at 250*g* for 10 min. Cell pellets were then resuspended in 1 mL of PBS, and this cell suspension was layered over 10 mL of Ficoll-Paque PLUS media (Cytiva 17144002) in a 15 mL centrifuge tube and centrifuged at 500*g* for 20 min in a swinging buck rotor to isolate PBMCs following the manufacturer’s instructions. Isolated PBMCs were resuspended in 5 mL of AIM V cell culture media (Fisher Scientific 31-035-025); aliquots were analyzed to determine viable cell concentrations by staining cells with a 0.4% Trypan Blue solution, and cell suspensions were adjusted to a final concentration of 3 × 10^6^/mL in AIM V cell culture media (Fisher Scientific 31-035-025), mixed with 40% fetal bovine serum and 20% dimethyl sulfoxide, and then stored in the vapor phase of a liquid nitrogen tank.

### PBMC Stimulation

Cryopreserved PBMC aliquots were rapidly thawed in a 37 °C water bath, mixed with an equal volume of RPMI-1640 media warmed to 37 °C, and then centrifuged at 400*g* for 5 min. Cell pellets were washed with 2 mL of RPMI-1640, resuspended in 150 μL of RPMI-1640, analyzed by Trypan Blue exclusion to evaluate cell viability, and then supplemented with RPMI-1640 to a final working concentration of ∼3 × 10^6^ viable cells/mL. Samples that had cell viabilities ≤70% were excluded from the analysis. PBMCs were plated in 6-well cell culture plates at a concentration of 1 × 10^6^ to 2 × 10^6^ viable cells/well as specified by different assay types and then stimulated with 10 ng/mL phorbol 12-myristate13-acetate (PMA, Sigma P1585) and 1 μg/mL ionomycin (STEM CELL 73722) or 1 μg/mL of the indicated peptide or peptide pools (Miltenyi Biotec 130-127-951) at 37 °C for the specified times.

### Flow Cytometry

PBMC aliquots suspended in AIM V cell culture media (2 × 10^6^/mL) were cultured overnight in 24-well culture plates before being stimulated for 24 h with PMA and ionomycin (10 ng/mL and 1 μg/mL, respectively) or a SARS-CoV-2 or HIV-p24 peptide pool (1 μg/mL), with 1 ng/mL IFN-γ transport blocker added 2 h after the start of induction. Following stimulation, PBMCs were pelleted by centrifugation at 500*g* for 5 min, PBS washed, then resuspended in 100 μL of IC fixation buffer and permeabilization buffer (eBioscience 00-8222-49 and 00-8333) for 10 min, and then incubated in a PBS/10% BSA solution supplemented with 1 μg/mL of an AlexaFluor488-labeled IFNγ-specific antibody (eBioscience 50-168-09) for 20 min. Flow cytometry analyses were performed using Attune flow cytometer (Thermo Scientific) gating cells, capturing the IFNγ-positive cell signal in the FITC/GFP channel, and analyzing and quantifying captured data with FlowJo software (v10.04).

### IGRA ELISAs

PBMCs (2 × 10^4^) were cultured for the indicated times at 37 °C in 0.1 mL of RPMI-1640 media supplemented with a 1 μg/mL SARS-COV-2 Spike peptide pool (Miltenyi Biotec 130-127-951), PMA and ionomycin (10 ng/mL and 1 μg/mL), or no added material, with an RPMI-only well included as a negative control. Culture supernatants were pipetted from each well and stored at −80 °C for future ELISA analysis.

After incubation, the media was pipetted from wells into a new 98 well plate. A 1 μg/mL final concentration of SARS-COV-2 peptide pool was added to the stimulation group. At 4, 6, 8, 10, 12, and 24 h, the supernatant was removed and stored at −80 °C for future ELISA.

IGRA ELISA plates were generated by incubating 96-well MaxiSorp plates (Nunc 44-2404-21) with 100 μL of 1 μg/mL PBS solution of human IFNγ-specific antibody (Invitrogen, M700-A) overnight at 4 °C. These plates were then washed 6 times with PBS/0.05% Tween 20 (PBST), blocked with 200 μL of 1% BSA/PBS for 1 h at room temperature, and then PBST washed, dried, and stored at 4 °C until use. Cryopreserved PBMC culture supernatant aliquots were thawed and transferred to assay plates in triplicate (50 μL/well) and incubated at room temperature for 1 h. 50 μL of IFNγ-biotin-labeled antibody (Invitrogen, M-701B) diluted at 1:1000 in 2% FBS/1× PBS was added to each well and incubated at room temperature for 1 h. Plates were washed and dried before pipetting 50 μL/well of poly-HRP streptavidin (Pierce, N200) diluted at 1:5000 in 1% BSA/1× PBS and incubated at room temperature for 30 min in the dark. Afterward, the plate was washed and dried for a final time. 100 μL/well of 3,3′,5,5′-tetramethylbenzidine (TMB, Thermo Scientific 34029) solution was added, and color development was observed. After adequate color development (∼10 min), 50 μL/well of stop solution (2.5 N H_2_SO_4_) was added, and plates were read at OD450.

### ELISpot

Filter screen plates (Millipore MAIPS4510) were coated with antihuman IFNγ (Invitrogen, M700-A, 1 mg/mL) at 1 μg/mL and stored overnight at 4 °C. The following day, the plate was washed 6 times with washing buffer (1× PBS + 1:2000 diluted Tween 20) and tapped dry. Wells were blocked with 200 μL of 1% BSA/1× PBS for 1 h at room temperature. 2 × 10^5^ PBMCs were then seeded into plates and stimulated with PMA-ionomycin (10 ng/mL and 1 μg/mL), SARS-CoV-2 Spike peptide pool (1 μg/mL), or HIV-p24 peptide (1 μg/mL). 100 μL of IFNγ-biotin-labeled antibody (Invitrogen, M-701B) diluted at 1:1000 in 1% BSA/1× PBS was added to each well and incubated at room temperature for 1 h. Plates were washed and dried before pipetting 100 μL/well of poly-HRP streptavidin (Pierce, N200) diluted at 1:5000 in 1% BSA/1× PBS and incubated at room temperature for 30 min in the dark. Then 100 μL/well of 3-amino-9-ethylcarbazole (AEC, BD 557630) was added and incubated at room temperature for 15 min. The whole plate was washed with deionized (DI) water, and the bottom was separated to be dried completely overnight. The spots were then scanned by a CTL-Immunospot S6 universal analyzer (ImmunoSpot) and counted by double-color ELISpot enzymatic software (ImmunoSpot).

### Chip Fabrication

The microfluidic design was fabricated on a silicon wafer using a conventional photolithography method that employed a negative photoresist,^59^ and the polydimethylsiloxane (PDMS) molds of the microfluidic device were generated on this silicon wafer (Figure S1). In this fabrication process, 20 g of PDMS elastomer was mixed with aliphatic amine at a 10:1 mass ratio and poured over the silicon wafer that contained three replicas of the device design, allowing the elastomer to cross-link and form the rigid structure of the microfluidic chip. PDMS solidification was accelerated by placing this mold in a 60 °C oven for 5 h, after which the three molds were removed from the silicon wafer for chip assembly. PDMS chip molds and 1 mm thick glass slides were then plasma treated to create silanol functional groups that formed strong covalent bonds to create the fluid-tight seals of the microfluidic channels. This chip was then incubated with 50 μg/mL polylysine (pH 7.4 in Tris-HCl buffer) for 30 min at 37 °C to form the polylysine nanolayer and washed with DI water to remove excess polylysine. Mean widths and heights of the resulting microfluidic channels were 400 and 100 μm, respectively, while the radii of the inlet, outlet, and capture chambers were 1.5, 3, and 3.5 mm, respectively.

### On-Chip ELISpot Assays

PMA-ionomycin (10 ng/mL and 1 μg/mL), SARS-CoV-2 Spike peptide pool (1 μg/mL), or HIV-p24 peptide (1 μg/mL) was added into 25 μL of whole blood and then incubated at 37 °C for 4 h. The blood samples were fixed with IC fixation buffer (eBioscience 00-8222-49) and permeabilization buffer (eBioscience 00-8333) at 25 °C for 20 min and then stained with 1 μg/mL anti-IFN-γ-Alexa488 (eBioscience 50-168-09) and 0.1 μg/mL Hoechst 33342 at 25 °C for 20 min. On-chip detection was performed as described above.

### Image Capture and Analysis

Images of the PBMCs attached microfluidic chamber were obtained using an EVOS M5000 imaging system, Invitrogen by Thermo Fisher Scientific, Madrid, Spain. Images (10×) of the stained PBMCs are representative of the total cell population and the IFNγ positive cells. The green fluorescence signal was obtained when Alexa 488 binds to intracellular IFNγ. The blue fluorescence signal from Hoechst 33342 represents the total cell counts. All the experiments were conducted in triplicate. Each time, four different random areas from the microfluidic chamber were chosen to obtain the images. All data acquired on the EVOS M5000 imaging system were analyzed using ImageJ software.

### Cell Counting

The total cell counts and IFNγ positive cell ratio were quantified using the National Institutes of Health (NIH) ImageJ image-analysis software. The images were converted to 8-bit grayscale. The lower threshold value was set to 70, and the higher threshold value was set to 255. The cell counts were analyzed with the size range from 1 to 100 (pixel^2^) and circularity 0.00–1.00.

## Supporting Information Available

Workflow PDMS microfluidic chip fabrication, including polylysine coating; AlexaFluor488-IFNγ, PE-tagged OX-40, and APC 4-1BB-tagged T-cell counting on glass surface; and flow cytometry gating of blood cells samples; correlation of on-chip IGRA results with traditional ELISpot and flow cytometry assays (PDF)

## Abbreviations

IGRA: interferon-gamma release assays
ELISpot: enzyme-linked immunosorbent spot
VOCs: variants of concern
RBC: red blood cell
PBMCs: peripheral blood mononuclear cells
PVDF: polyvinylidene difluoride
IFNγ: interferon gamma
