# Supplementary material for: Evaluation of SARS-CoV-2-Specific T-Cell Activation with a Rapid On-Chip IGRA
Source: ACS Nano. 2023 Jan 3;17(2):1206–16. doi: 10.1021/acsnano.2c09018 (PMC9878992; doi:10.1021/acsnano.2c09018)
Supplement: Supplementary file 1 — nn2c09018_si_001.pdf [file nn2c09018_si_001.pdf]

Supplementary Information

Evaluation of SARS-CoV-2 specific T cell activation with a rapid on-chip IGRA

Bo Ning[1,2], Sutapa Chandra[1,2], Juniper Rosen[1,2], Evan Multala[1,2], Melvin Argrave[1,2], Lane Pierson[1,2], Ivy Trinh[3], Brittany Simone[4], Matthew David Escarra[4], Stacy Drury[5,6], Kevin J. Zwezdaryk[3], Elizabeth Norton[3] Christopher J. Lyon[1,2], and Tony Hu[1,2*]*

[1]Center for Cellular and Molecular Diagnostics, Tulane University School of Medicine, New Orleans, LA 70112, USA

[2]Department of Biochemistry and Molecular Biology, Tulane University School of Medicine, New Orleans, LA 70112, USA

[3]Department of Microbiology & Immunology, Tulane University School of Medicine, New Orleans, LA70112, USA

[4]Department of Physics and Engineering Physics, Tulane University, New Orleans, LA 70118, USA

[5]Department of Psychiatry, Tulane University, New Orleans, LA, 70112, USA

[6]Tulane Brain Institute, Tulane University, New Orleans, LA 70112, USA

*Corresponding to: Tony Hu, Tonyhu@tulane.edu

Bo Ning, Bning1@tulane.edu

Keywords: T cell response, COVID-19, IGRA, COVID-19 vaccine, rapid test, whole blood assay

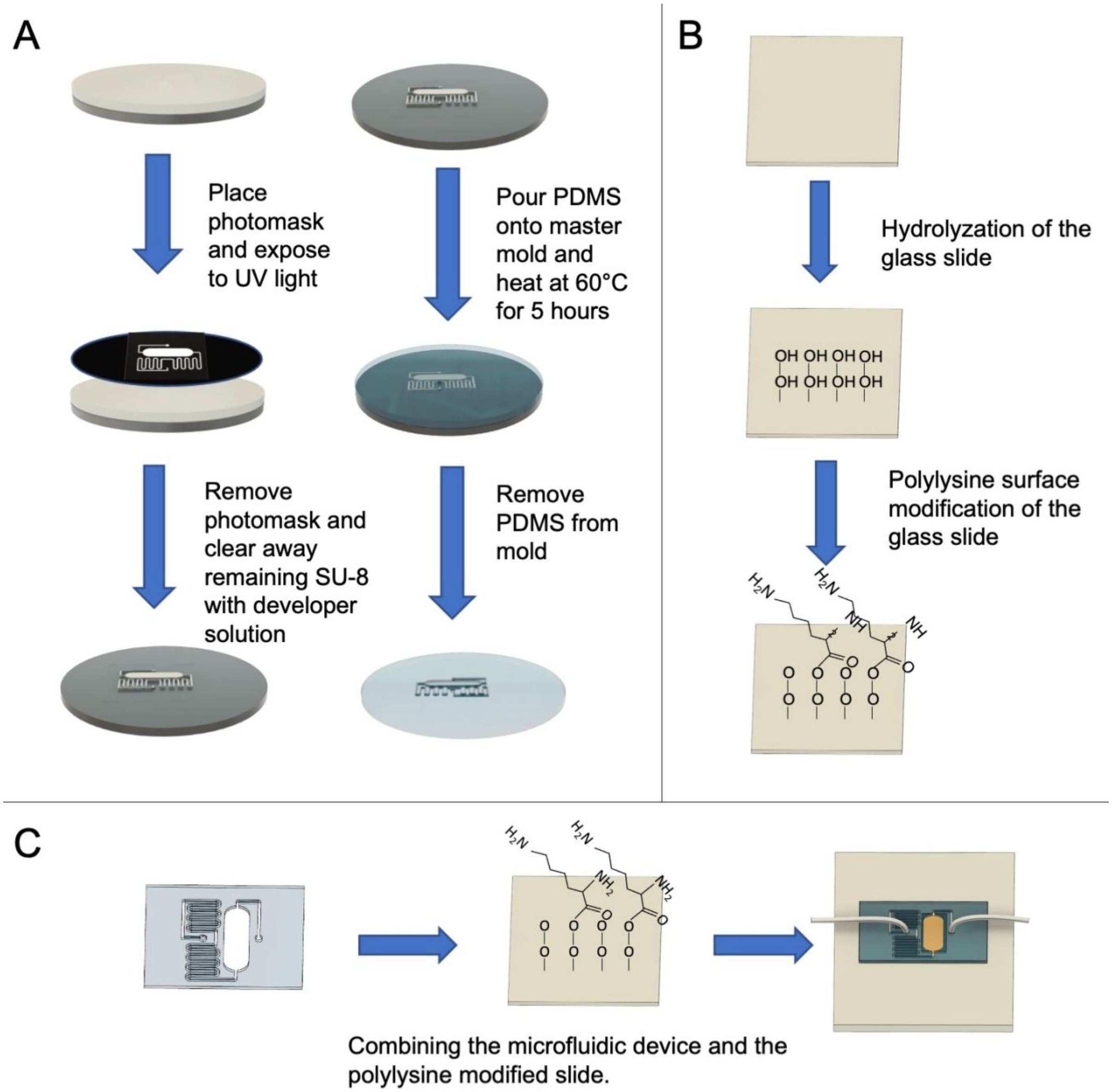

Figure S1. PDMS microfluidic chip fabrication. (**A**) Silicon wafers coated with SU8 Epoxy are then covered with a photomask containing the device design, exposed to ultraviolet light, and washed with SU8 developer to remove inactivated SU8 surrounding the device design. Then a 10:1 PDMS-to-curing agent mixture is poured onto the master wafer and then heated at 60°C for 5 h. (**B**) 50 µg/ml polylysine (pH 7.4) was coated on glass surface for 30 min and washed with DI water to form the nanolayer. (**C**) Solidified PDMS device is cut from the wafer and bound to the polylysine coated glass slide previously exposed to oxygen plasma to generate the complete device.

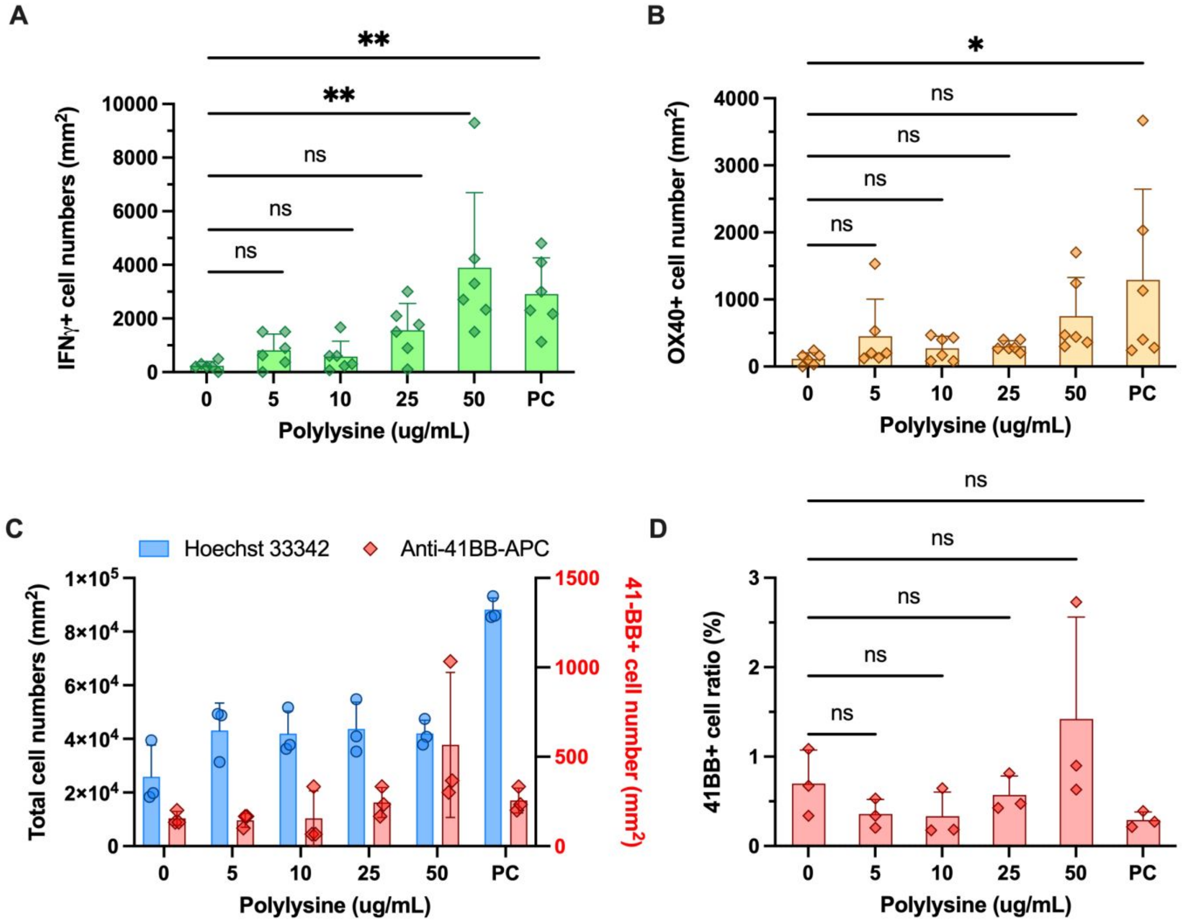

Figure S2. Activated T cell counting on glass surface. PBMC capture on well coated with and without different polylysine concentrations, seeded with $2{\times}10^5$ PBMCs, induced with PMA/ionomycin for 4 h, and stained with Hoechst 33342 and incubated with AlexaFluor488- IFNγ (**A**), PE-tagged OX-40 (**B**) or APC tagged 4-1BB (**C**&**D**) specific antibodies, respectively. Wells were analyzed for activated T cell counts using a fluorescent plate reader. Positive control (PC) wells indicate signal detected in wells that were not washed to remove non- or weakly adherent cells.

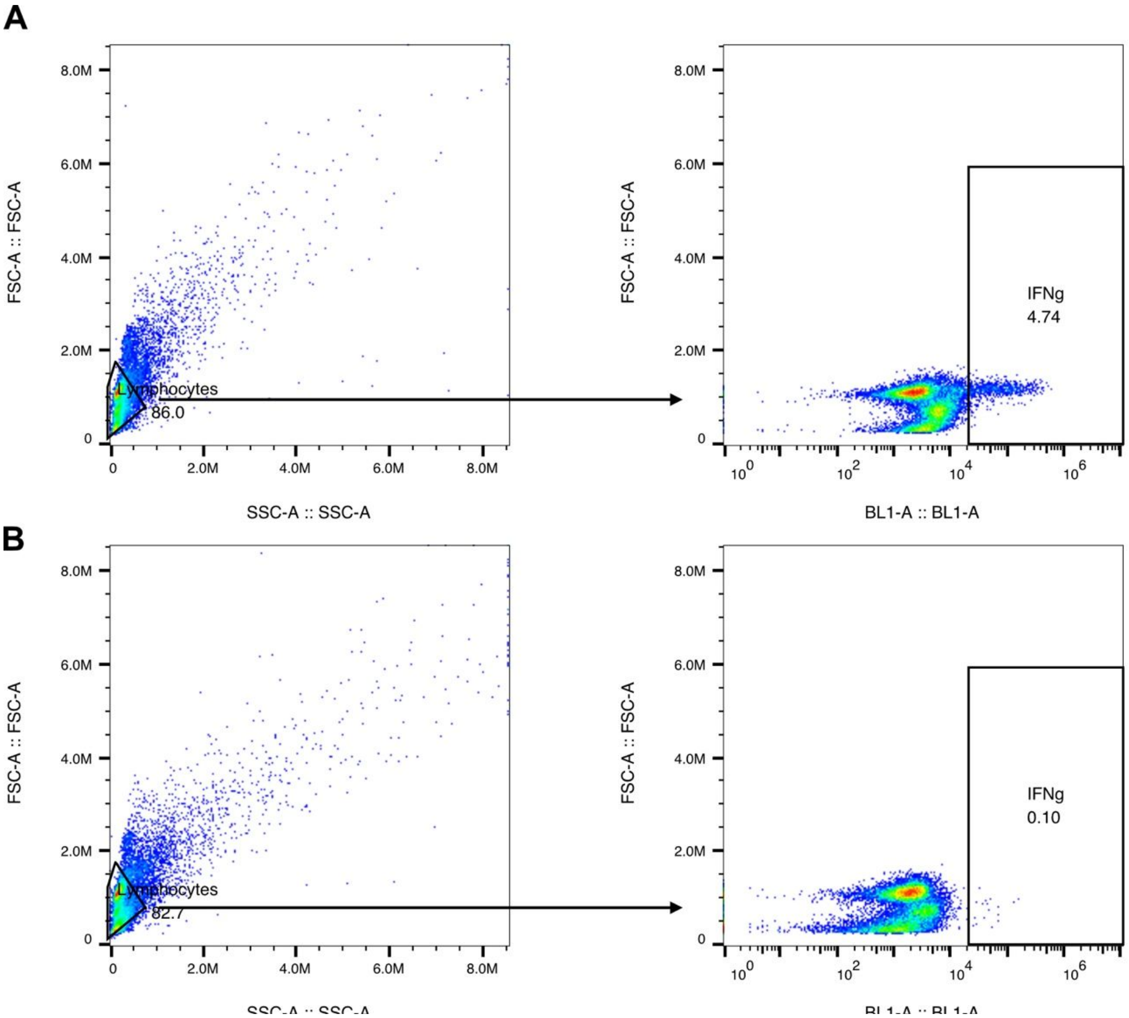

Figure S3. Flow cytometry gating of blood cells samples. Example of the IFNγ response detected upon flow cytometry analysis of ~2×10^6 PBMCs isolated from HIV-negative SARS-CoV-2-vaccinated (3 doses) after 24 h exposure to (**A**) SARS-CoV-2 spike peptides or (**B**) HIV-1 p24 (non-specific control) peptides. Scatterplots indicate the total PBMC scattering and lymphocyte gate (left panels) and the distribution of the IFNγ-negative and IFNγ-positive (gated population) in the lymphocytes gate (right panels).

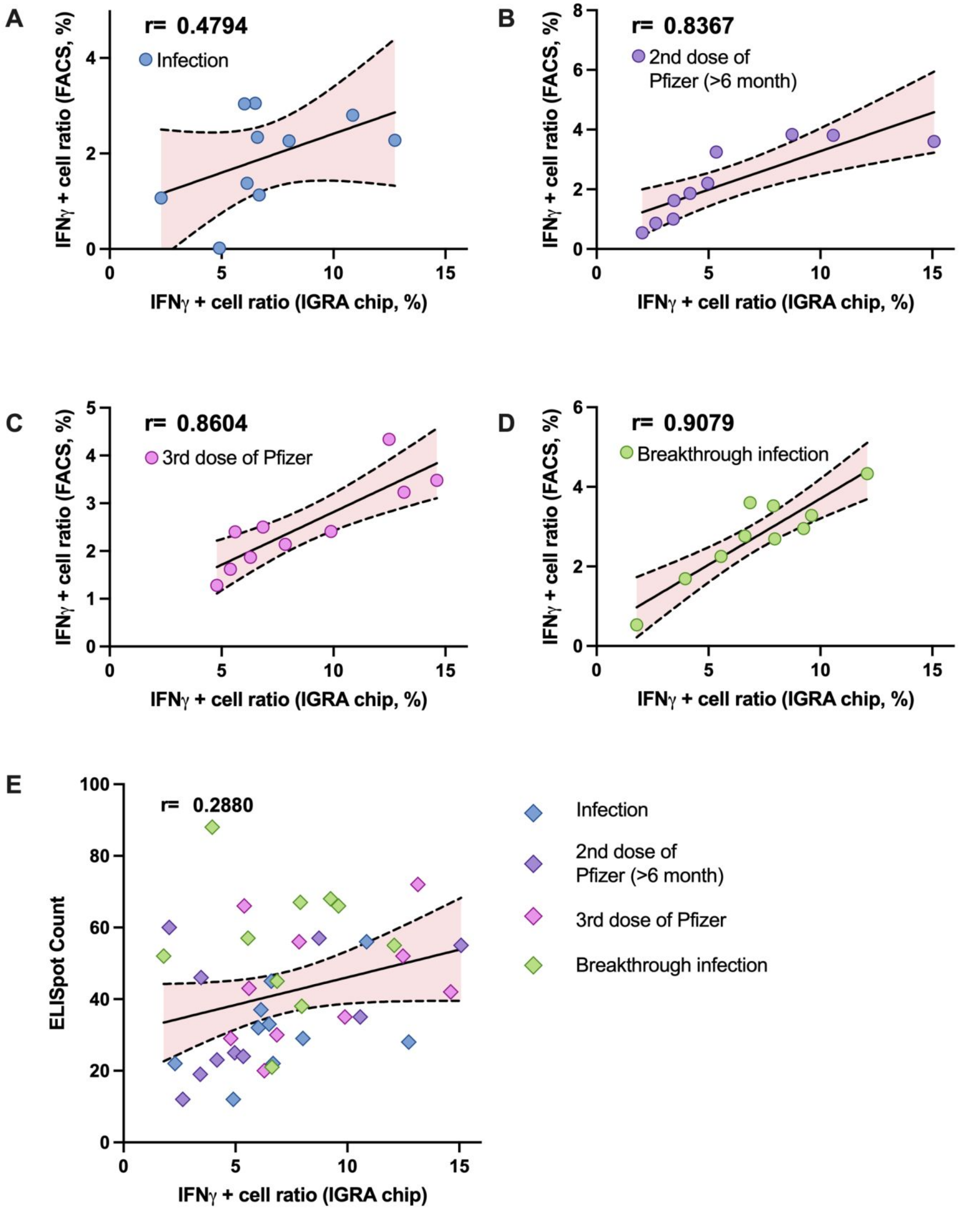

Figure S4. Correlation of On-Chip IGRA results with traditional assays. (**A-D**) Correlations of on-chip ELISpot and flowcytometry results among the (**A**) SARS-Cov-2, (**B**) second and (**C**) third vaccine dose groups and (**D**) the vaccinated individuals with breakthrough infections. (**E**) Correlation of on-chip ELISpot assay and standard ELISpot assay results. Data indicate Spearmann/Pearson r-values.
